# Sleep quality and air conditioner use

**DOI:** 10.1186/2046-7648-4-S1-A129

**Published:** 2015-09-14

**Authors:** Kazuyo Tsuzuki, Naomi Morito, Hajime Nishimiya

**Affiliations:** 1 National Institute of Advanced Industrial Science and Technology, Tsukuba, Japan

## Introduction

Airflow is an effective way to increase heat loss - an ongoing process during sleep and wakefulness in daily life. In a previous study, alleviation of heat by increased isothermal airflow reduced wakefulness, skin temperature, rectal temperature, and sweating during sleep at 32 °C with 80% relative humidity [1]. In this study, experiments were conducted to determine the effect on sleep of varying airflow velocity from air conditioners, using 10 healthy young men (age 23.0 +/- 3.6 years; height 170.9 +/- 3.6cm; mass 62.2 +/- 6.8kg) as subjects.

## Methods

The desired environmental conditions were attained by using a normal air conditioner fastened to the wall in one room and a new ceiling air conditioner in another room. The ceiling-mounted air conditioner dispersed airflow from a 2-piece ceiling panel (1,800 × 900 mm) covered with a 3-dimensional knit fabric. Electroencephalography, electrooculography, heart rate, skin temperature, and rectal temperature of the subjects were continuously measured during the experiments. A questionnaire for thermal sensation and comfort (bipolar 7 point scale), sleepiness, and sleep quality [2] was used before and after sleep.

## Results

The mean air temperature, relative humidity, and air velocity in the rooms with the wall-mounted air conditioner were 26.4 °C, 58 %, and 0.14 m.s^-1^, respectively, and 26.4°C, 53 %, and 0.04 m.s^-1^, respectively, with the ceiling-mounted air conditioner. The maximum air velocity above the bed in the rooms and the frequency of airflow from the wall-mounted air conditioner were 1.1 m.s^-1^and 28 times per night, respectively, and 0.3 m.s^-1^and 11 times per night, respectively, with the ceiling-mounted air conditioner. The comfort sensation (+1.5 vs. +2: comfortable) and the total duration of each sleep stage, as determined by the sleep efficiency index (92.1% vs. 92.6 %), did not differ significantly with the two air conditioners. However, the subjects felt a stronger draft and slightly colder due to faster velocity and higher frequency of airflow. The skin temperature on the forehead and uncovered arm and hand decreased due to higher airflow. We found that subjects sleeping under higher airflow had an increased number of wakings, increased heart rates, and greater body movement. The subject was assumed to move to avoid airflow from the air conditioner. To determine which sleep stage was most affected by airflow we compared those parameters. More number waking events (0.23 vs 0.05, p < 0.01) due to airflow were observed in light (stages 1 + 2) sleep compared to slow-wave (stages 3 + 4) sleep (SWS), but increase in heart rate (0.32 vs 0.23, p = 0.24) and body movement (0.61 vs 0.51, p = 0.48) were similar between light sleep and SWS.

## Discussion

A previous study reported that cooling due to temperature decrease is more disruptive to Rapid eye movement (REM) sleep than SWS (light sleep was not examined) [3]. Our result suggests that light sleep is especially susceptible to being disrupted than SWS by cool airflow. Moreover, decreases in skin temperature and marked increases in heart rate due to body movements were observed in both light sleep and SWS.

## Conclusion

These results indicate that cool airflow from an air conditioner should not be directed at sleeping subjects.
